# Survival outcomes of HIV-positive adults on peritoneal dialysis at Helen Joseph renal unit

**DOI:** 10.4102/sajhivmed.v24i1.1471

**Published:** 2023-05-10

**Authors:** Kagisho L. Thomas, Malcolm Davies

**Affiliations:** 1 Department of Internal Medicine, Faculty of Health Sciences, University of the Witwatersrand, Johannesburg, South Africa; 2 Renal Department, Faculty of Health Sciences, University of the Witwatersrand, Johannesburg, South Africa

**Keywords:** HIV, continuous ambulatory peritoneal dialysis, chronic kidney disease, peritonitis, antiretrovirals

## Abstract

**Background:**

HIV is a risk factor for the development of chronic kidney disease. People with chronic kidney disease in the state sector are likely to be prescribed continuous ambulatory peritoneal dialysis (CAPD). Previous studies have raised concern about the safety of CAPD in people living with HIV (PLWH) compared to HIV-negative patients.

**Objectives:**

To compare the risk of peritonitis, and modality and patient survival by HIV status in patients receiving CAPD at Helen Joseph Hospital.

**Method:**

A retrospective study of patients receiving CAPD between 01 January 2007 and 31 December 2017 was undertaken. Five-year patient and modality survival were modelled for PLWH and HIV-negative subgroups and analysed using the log-rank test; the effect of CD4 count, HIV viral load, and duration of antiretroviral therapy on these parameters in PLWH were additionally modelled using the Cox Proportional Hazards technique.

**Results:**

Eighty-four patients, comprising of 21 PLWH and 63 HIV-negative patients, were analysed. No difference was observed in the proportion of patients who had at least one episode of peritonitis between PLWH (61.2%) and HIV-negative patients (63.5%) (*P* = 0.547). A trend towards increased risk of peritonitis due to Gram-negative organisms in PLWH was noted (odds ratio: 3.20, 95% confidence interval: 0.86–11.9, *P* = 0.083). No difference was observed in 5-year patient or modality survival on CAPD between PLWH (log-rank *P* = 0.161) and HIV-negative patients (log-rank *P* = 0.240).

**Conclusion:**

People living with HIV should not be excluded from CAPD as a mode of kidney replacement therapy (KRT).

**What this study adds:** Patients with HIV should not be excluded from continuous ambulatory peritoneal dialysis kidney replacement therapy.

## Introduction

South Africa is the epicentre of the global HIV epidemic, with an estimated overall prevalence rate of 13.9%, and approximately 8.45 million people living with the virus as of 2022.^1^ The advent of the antiretroviral therapy (ART) programme in 2004 decreased AIDS-related deaths from 345 185 in 2006 to 126 755 in 2017, and resulted in an improvement in life expectancy from 52.2 years to 61.2 years in male patients, and 55.3 years to 66.7 years in female patients.^1^ These victories in survival in people living with HIV (PLWH) may, however, come at the cost of increases in the burden of non-communicable disease, including chronic kidney disease (CKD).

HIV infection has long been recognised as an aetiological factor in kidney disease.^2^ In populations of recent African ancestry, genetic polymorphisms in the apolipoprotein-1 protein coding sequence has been associated with a higher prevalence of CKD due to increased risk of the development of HIV-associated nephropathy.^3^ Apolipoprotein-1 mutations are also known to increase the risk of focal segmental glomerulosclerosis and hypertensive nephropathy, and may also play a role in the progression of diabetic kidney disease.^2,4^ In addition, exposure to nephrotoxic agents including ART and prophylactic antibiotics, as well as opportunistic infections and hepatitis B and C co-infections increases the risk of kidney disease in PLWH.^2,3^

Improved survival in PLWH due to widespread availability of ART increases the probability of the development of age- and lifestyle-related disorders such as hypertension and diabetes.^5,6^ Furthermore, relative normalisation of immune system function in patients taking ART increases the possibility of the development of immune-mediated kidney disease such as focal segmental glomerulosclerosis.^4^ These considerations underlie the observation that, despite a decrease in HIV-associated nephropathy incidence since ART rollout, there has been an increase in CKD in PLWH.^3,7^ Improved life expectancy in the HIV and/or AIDS population further increases the probability of progression of CKD requiring long-term kidney replacement therapy (KRT).^1,2^

South Africa’s historical inequalities mean that PLWH who develop CKD are most likely to access KRT through the public state sector. Chronic resource limitations in state dialysis units limit individual patient choice as to the dialysis modality offered to new initiates. Reduced staff and infrastructure costs, and lowered patient transport costs,^8,9^ result in many state dialysis units preferably initiating onto continuous ambulatory peritoneal dialysis (CAPD) rather than haemodialysis. As a result, CAPD is more frequently prescribed in state units than is the case in the private sector.^10^

Patient outcomes on CAPD in general are similar to those receiving haemodialysis^10,11^ in high-income countries, with a paucity of data in low-income countries. However, some concern exists as to the safety of CAPD in PLWH, with a previous South African series suggesting an increased risk of peritonitis and modality failure in this group.^12^ These risks appear to relate to the severity of immunodeficiency in PLWH initiating CAPD, which in turn suggests that ART prescription may ameliorate this risk. Since institutional policy at the Helen Joseph Dialysis Unit requires ART initiation before CAPD prescription, we investigated the effect of HIV-positive status on patient and modality outcomes in a cohort of patients initiating dialysis between 2007 and 2017.

## Research methods and design

Helen Joseph Hospital is a tertiary-level facility which provides KRT to patients resident in the western areas of Johannesburg in the Gauteng province. The hospital pursues a ‘CAPD first’ policy. People living with HIV do not need to demonstrate virological control prior to dialysis but must have been started on ART prior to CAPD initiation. In addition, PLWH on dialysis are referred to the on-site HIV clinic for optimal management.

All patients above the age of 18 years initiating CAPD at the Helen Joseph Hospital between 01 January 2007 and 31 December 2017 were considered for inclusion; patients with missing medical records were excluded from the study. Consecutive sampling was used to identify patients for inclusion and the final data set comprised all patients on CAPD during the study period who met inclusion criteria. The patients had to be on dialysis for at least 3 months to be considered eligible for the study. There were no patients on automated peritoneal dialysis enrolled in the study.

Anonymised data including patient demographics (age, gender and ethnicity), comorbidities (diabetes mellitus, hypertension and known cardiovascular disease), HIV-positive status (including CD4 count, viral load and duration of ART prior to dialysis initiation), HIV-associated nephropathy (defined as CKD in PLWH with no apparent aetiology persisting for more than 3 months, presenting with sub-nephrotic proteinuria and/or enlarged kidneys on ultrasound), peritonitis episodes and aetiological organism, and patient and CAPD modality survival data were extracted from clinical records and stored in an Excel^®^ database which was subsequently exported for analysis using Statistica version 14 (Tibco software, Palo Alto, California, United States).

Distribution of continuous data was assessed using the Shapiro-Wilk W test and through visual inspection of the histogram plot. The proportions experiencing at least one episode of peritonitis, and microbiology of peritonitis episodes, were described and compared between PLWH and HIV-negative patients using the Pearson Chi-square test. Time to first episode of peritonitis from modality initiation was compared between PLWH and HIV-negative patients using the Mann-Whitney U test. The effect of CD4 count, HIV viral load and duration of ART prescription at CAPD initiation on time to first episode of peritonitis were modelled in PLWH using Cox Proportional Hazards modelling. Five-year patient and modality survival from time of CAPD initiation were modelled for PLWH and HIV-negative subgroups and analysed using the log-rank test; the effect of CD4 count, HIV viral load and duration of ART on these parameters in PLWH were additionally modelled using the Cox Proportional Hazards technique.

### Ethical considerations

The study was approved by the University of the Witwatersrand Human Research Ethics Committee (protocol number M190506).

## Results

Of 115 patients initiated onto CAPD during the study period, 31 were excluded due to incomplete data, resulting in 84 patients, comprising 21 PLWH and 63 patients who were HIV-negative, being included in the final analysis. Baseline characteristics of the cohort are shown in Table 1.

**TABLE 1:** Baseline characteristics of patients receiving continuous ambulatory peritoneal dialysis Helen Joseph Hospital, 2007–2017.

Baseline characteristics	All (*n* = 84)				PLWH (*n* = 21, 25%)				HIV-negative (*n* = 63, 75%)				*P*
	Median	Interquartile range	*n*	%	Median	Interquartile range	*n*	%	Median	Interquartile range	*n*	%	
**Age at CAPD initiation (years)**	42.5	32.5–50.5	-	-	41	35–47	-	-	44	30–52	-	-	0.735
**Gender**													0.131
Female	-	-	39	46.4	-	-	13	61.9	-	-	26	41.3	
Male	-	-	45	53.6	-	-	8	38.1	-	-	37	58.7	
**Ethnicity**													0.094
Black African patients	-	-	57	67.8	-	-	19	90.5	-	-	38	60.3	
Mixed race patients	-	-	14	16.7	-	-	1	4.7	-	-	13	20.6	
White patients	-	-	9	10.7	-	-	0	0.0	-	-	9	14.3	
Asian patients	-	-	3	3.6	-	-	1	4.7	-	-	2	3.2	
Indian patients	-	-	1	1.2	-	-	0	0.0	-	-	1	1.6	
**Comorbid diabetes**	-	-	14	16.7	-	-	3	14.3	-	-	11	17.5	0.516
**Comorbid hypertension**	-	-	79	94.1	-	-	21	100.0	-	-	58	92.1	0.327
**Known comorbid cardiovascular disease**	-	-	4	4.8	-	-	1	4.7	-	-	3	4.7	0.691
**Ascribed aetiology of kidney failure**													
Hypertension	-	-	60	71.4	-	-	10	47.6	-	-	50	79.4	
HIV-associated kidney disease	-	-	9	10.7	-	-	9	42.9	-	-	-	-	
Diabetic kidney disease	-	-	8	9.5	-	-	1	4.7	-	-	7	11.1	
Other glomerular disease	-	-	6	7.1	-	-	1	4.7	-	-	5	7.9	
Polycystic kidney disease	-	-	1	1.2	-	-	0	0.0	-	-	1	1.6	
**ART regimen and HIV infection control status in PLWH receiving CAPD at HJH**													
**Prescribed ART regimen**													
Efavirenz-based regimen	-	-	20	95.2	-	-	-	-	-	-	-	-	-
Lopinavir-based regimen	-	-	1	4.8	-	-	-	-	-	-	-	-	-
**Duration of ART prescription before CAPD initiation (months)**	16	12–39	-	-	-	-	-	-	-	-	-	-	-
**CD4 count at CAPD initiation (×106/mm^3^)**	320	199–425	-	-	-	-	-	-	-	-	-	-	-
**Number of patients with CD4 count > 200 × 106/mm^3^ cells at CAPD initiation**	-	-	14	66.7	-	-	-	-	-	-	-	-	-
**HIV viral load at CAPD initiation (copies/mL)**	70	25–327	-	-	-	-	-	-	-	-	-	-	-
**Number of patients with viral load < 50 copies/mL at CAPD initiation**	-	-	8	38.1	-	-	-	-	-	-	-	-	-

PLWH, people living with HIV; ART, antiretroviral therapy; CAPD, continuous ambulatory peritoneal dialysis; HJH, Helen Joseph Hospital.

All PLWH in this study were initiated onto ART prior to CAPD start, although there was considerable variation in the duration of ART preceding dialysis (range 1–108 months). Among patients not yet achieving virological control on ART, the median viral load was 249 copies/mL (range 55 copies/mL – 110 000 copies/mL).

A total of 111 episodes of peritonitis were recorded among 53 patients. Forty (63.5%) HIV-negative patients experienced at least one episode of peritonitis compared to 13 (61.2%) PLWH (*P* = 0.547). A total of 84 episodes of peritonitis were recorded in HIV-negative patients compared to 27 in PLWH; the proportions of peritonitis episodes between HIV-negative patients and PLWH was not significantly different (*P* = 0.943). There was no significant difference in the median time to first episode of peritonitis between PLWH (12.7 months) and HIV-negative (10.4 months) patients in this series (*P* = 0.125).

A large proportion of all peritonitis episodes in this cohort were culture negative (46 cases, 41.4%); rates of culture-negative peritonitis were not significantly different between PLWH (12 cases, 26.1%) and HIV-negative (34 cases, 73.9%) patients (*P* = 0.823) (Table 2). Gram-positive bacteria were the most common isolates (31 cases, 47.7%) among culture-positive peritonitis episodes, followed by Gram-negative (28 cases, 43.1%); fungal peritonitis contributed six cases (9.2%) and there were no cases of tuberculous peritonitis in this cohort. Gram-positive organisms were more frequent isolates in HIV-negative patients (54% of all cultures organisms in this group), while Gram-negatives were more frequently cultured in PLWH (60%); a trend towards increased odds of culturing Gram-negative organisms in PLWH with bacterial peritoneal dialysis-associated peritonitis was observed (odds ratio [OR]: 3.20, 95% confidence interval [CI]: 0.86–11.9, *P* = 0.083).

**TABLE 2:** Peritoneal dialysis-associated peritonitis.

Variable	All				People living with HIV				HIV-negative			
	Mean	Interquartile range	*n*	%	Mean	Interquartile range	*n*	%	Mean	Interquartile range	*n*	%
Time to first episode of peritonitis (months)	12.0	5.5–23.2	-	-	10.4	3.1–16.8	-	-	12.7	5.7–25.9	-	-
Number of episodes of peritonitis	2	1–3	-	-	2	1–2	-	-	2	1–3	-	-
**Isolated organism**												
Culture negative	-	-	46	41.4	-	-	12	26.1	-	-	34	73.9
Organism isolated on culture	-	-	65	58.6	-	-	15	55.5	-	-	50	59.5
Gram-positive isolates	-	-	31	47.7	-	-	4	26.7	-	-	27	54.0
*Staphylococcus epidermidis*	-	-	15	23.8	-	-	3	20.0	-	-	12	25.0
*Staphyloccus aureus*	-	-	5	7.9	-	-	0	0.0	-	-	5	10.4
*Staphyloccus haemolyticus*	-	-	4	9.4	-	-	0	0.0	-	-	4	8.3
*Corynebacterium* spp.	-	-	2	3.2	-	-	0	0.0	-	-	2	4.2
*Streptoccus oralis*	-	-	1	1.6	-	-	0	0.0	-	-	1	2.1
*Micrococcus* spp.	-	-	1	1.6	-	-	0	0.0	-	-	1	2.1
*Streptococcus cristatus*	-	-	1	1.6	-	-	0	0.0	-	-	1	2.1
*Streptococcus mitis*	-	-	1	1.6	-	-	1	6.7	0		0	0.0
*Streptococcus pneumoniae*	-	-	1	1.6	-	-	0	0.0	-	-	1	2.1
Gram-negative isolates	-	-	28	43.1	-	-	9	60.0	-	-	19	38.0
*Escherichia coli*	-	-	8	12.7	-	-	2	13.3	-	-	6	12.5
*Klebsiella pneumoniae*	-	-	6	9.5	-	-	4	26.7	-	-	2	4.2
*Enterobacter cloacae*	-	-	5	7.9	-	-	1	6.7	-	-	4	8.3
*Pseudomonas aeruginosa*	-	-	3	4.8	-	-	1	6.7	-	-	19	38.0
*Klebsiella aerogenes*	-	-	1	1.6	-	-	1	6.7	0		0	0.0
*Serratia marascens*	-	-	1	1.6	-	-	0	0.0	-	-	1	2.1
*Citrobacter amalonaticus*	-	-	1	1.6	-	-	0	0.0	-	-	1	2.1
*Campylobacter* spp.	-	-	1	1.6	-	-	0	0.0	-	-	1	2.1
*Acinetobacter* spp.	-	-	1	1.6	-	-	0	0.0	-	-	1	2.1
*Aeromonas* spp.	-	-	1	1.6	-	-	0	0.0	-	-	1	2.1
*Fungal*	-	-	6	9.2	-	-	2	13.3	-	-	4	8.0
*Candida parapsilosis*	-	-	3	4.8	-	-	1	6.7	-	-	2	4.2
*Cryptococcus neoformans*	-	-	1	1.6	-	-	1	6.7	-	-	-	-
Fungal organism not identified	-	-	-	-	-	-	-	-	-	-	2	4.2

**FIGURE 1:**
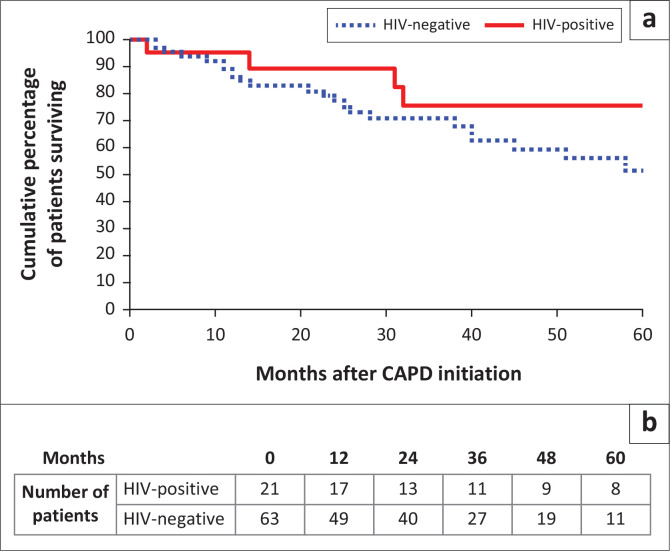
Patient survival at 5 years of follow-up.

**FIGURE 2:**
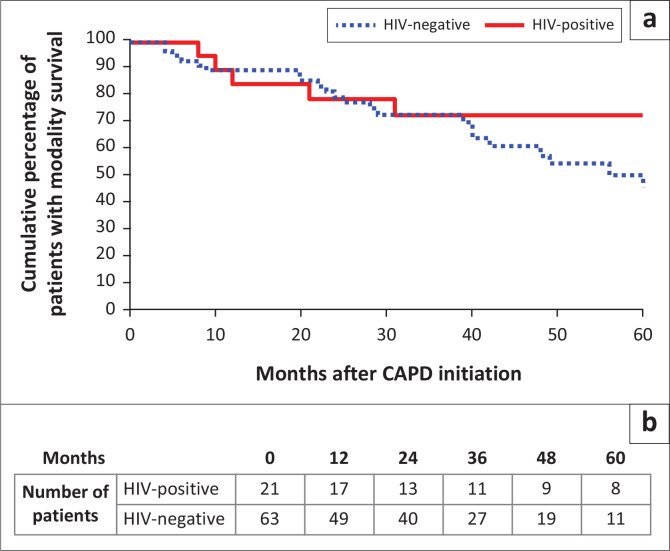
Modality survival at 5 years of follow-up.

Overall patient survival on CAPD among PLWH was 76.2% compared to 60.3% for HIV-negative patients (*P* = 0.292); modality survival among PLWH was 71.4% compared to 61.9% for the HIV-negative cohort (*P* = 0.599). Five-year patient (Figure 1) and modality (Figure 2) survival were not significantly different between HIV-positive subgroups (log-rank *P* = 0.153 and *P* = 0.233, respectively).

No effect was detected for CD4 count, HIV viral load, or duration of ART in PLWH on either the time to first episode of peritonitis (*P* = 0.602, *P* = 0.723, and *P* = 0.164, respectively), 5-year patient survival (*P* = 0.953, *P* = 0.238, and *P* = 0.635, respectively) or on 5-year modality survival (*P* = 0.427, *P* = 0.783, and *P* = 0.310, respectively).

## Discussion

This study provides evidence for the safety of CAPD in PLWH in the context of universal access to ART. In particular, PLWH do not appear to be at greater risk of peritonitis, and patient and modality survival on CAPD appear to be similar to that of HIV-negative patients.

The need for improved access to KRT among PLWH is well illustrated by the observation that 25% of patients included in this cohort were HIV-positive, compared to the estimated population prevalence of HIV infection in South Africa of 12.6% during the study period.^1^ Continuous ambulatory peritoneal dialysis offers an attractive means to increase KRT availability in the South African context, but concerns exist about the safety of this modality in PLWH. In particular, an increased risk of CAPD-related peritonitis has been reported in PLWH who have CD4 counts below 200 cells/mm^3^ at dialysis initiation.^13^ In the present study, HIV infection did not increase the number of peritonitis episodes, and no effect was observed for HIV positivity, viral load or CD4 count on time to first episode of peritonitis. Reductions in peritonitis risk in the present cohort are likely mediated by use of ART, as a result of which two-thirds of PLWH included in this series had a CD4 count at dialysis initiation above 200 cells/mm^3^.

While overall risk for peritonitis may be independent of HIV status, seropositivity may affect the microbiological pattern of causative organisms encountered. Previous South African reports have suggested an increased risk of *Staphylococcus* species in the nasal carriage, with lower CD4 count in PLWH on CAPD, which has been proposed as a risk factor for peritonitis.^14^ In contrast, the present study found a trend towards increased odds of Gram-negative peritonitis in PLWH. A growing body of literature reports intestinal dysbiosis in PLHW, which may not revert even with successful virological suppression on ART.^15^ This persistent dysbiosis may result in chronic gut wall inflammation,^16^ in turn facilitating transmural translocation,^15^ a known pathogenic pathway for the development of Gram-negative CAPD-related peritonitis.^17^

Peritonitis has been reported to be the most important modifiable risk factor for modality survival.^18^ Similar peritonitis risk between PLWH and HIV-negative patients through universal ART prescription in the present study is likely to have been a significant contributor to survival outcomes in PLWH in this series. However, additional factors related to the selection of patients for CAPD as practised at this institution are also likely to have played a role. In particular, restriction of CAPD access to those patients with home circumstances favourable to the creation of a suitably sterile environment to perform dialysate indwell catheterisation is likely to have contributed to reductions in peritonitis rates.

Peritonitis is also known to be an independent contributor to survival of patients on CAPD.^19^ Reduction in peritonitis risk in PLWH on ART may well have contributed to improved survival in the present cohort; in addition, universal access to ART has been shown to directly increase survival in PLWH prescribed either haemo- or peritoneal dialysis.^20^

There are several limitations to this study. The retrospective nature of this study resulted in the exclusion of a significant number of patients, which may have led to sample bias. Furthermore, the retrospective methodology employed limited the ability to include other parameters, such as socio-economic status and education level, which are known to contribute to survival on CAPD. The small sample size especially limited the generalisation of the results from the patient and modality survival analyses. Finally, the single-centre nature of this study may limit generalisability of its findings. It should, however, be noted that restriction of this study to a single centre ensured homogeneity of patient selection for CAPD as well as HIV infection management, which in turn may have reduced the risk of error.

## Conclusion

People living with HIV constitute a significant proportion of patients developing dialysis-requiring kidney failure in South Africa. Risk of peritonitis was not increased and modality and patient survival were not poorer in PLWH on CAPD. Peritoneal dialysis appears to be a safe KRT in the era of universal access to ARTs.
